# Effects of Seated Postural Stability and Trunk and Upper Extremity Strength on Performance during Manual Wheelchair Propulsion Tests in Individuals with Spinal Cord Injury: An Exploratory Study

**DOI:** 10.1155/2016/6842324

**Published:** 2016-08-18

**Authors:** Dany H. Gagnon, Audrey Roy, Sharon Gabison, Cyril Duclos, Molly C. Verrier, Sylvie Nadeau

**Affiliations:** ^1^Pathokinesiology Laboratory, Centre for Interdisciplinary Research in Rehabilitation of Greater Montreal, Institut de Réadaptation Gingras-Lindsay-de-Montréal (IRGLM), Montreal, QC, Canada H3S 2J4; ^2^School of Rehabilitation, Université de Montréal, Montreal, QC, Canada H3C 3J7; ^3^Toronto Rehabilitation Institute, University Health Network, Lyndhurst Centre, Toronto, ON, Canada M4G 3V9; ^4^Department of Physical Therapy, Faculty of Medicine, University of Toronto, Toronto, ON, Canada M5G 1V7

## Abstract

*Objectives.* To quantify the association between performance-based manual wheelchair propulsion tests (20 m propulsion test, slalom test, and 6 min propulsion test), trunk and upper extremity (U/E) strength, and seated reaching capability and to establish which ones of these variables best predict performance at these tests.* Methods.* 15 individuals with a spinal cord injury (SCI) performed the three wheelchair propulsion tests prior to discharge from inpatient SCI rehabilitation. Trunk and U/E strength and seated reaching capability with unilateral hand support were also measured. Bivariate correlation and multiple linear regression analyses allowed determining the best determinants and predictors, respectively.* Results.* The performance at the three tests was moderately or strongly correlated with anterior and lateral flexion trunk strength, anterior seated reaching distance, and the shoulder, elbow, and handgrip strength measures. Shoulder adductor strength-weakest side explained 53% of the variance on the 20-meter propulsion test-maximum velocity. Shoulder adductor strength-strongest side and forward seated reaching distance explained 71% of the variance on the slalom test. Handgrip strength explained 52% of the variance on the 6-minute propulsion test.* Conclusion.* Performance at the manual wheelchair propulsion tests is explained by a combination of factors that should be considered in rehabilitation.

## 1. Introduction

Many individuals who have sustained sensorimotor impairments that challenge their walking ability will use a manual wheelchair as their primary means of mobility. Consequently, their upper extremities (U/E), especially their shoulders, are exposed to repetitive movements coupled with elevated demands that may contribute to the high incidence of secondary U/E musculoskeletal impairments in this population. During manual wheelchair propulsion, numerous kinetic [1–5], electromyographic [6–10], and musculoskeletal modelling [11] studies have confirmed substantial solicitation of key shoulder muscles (i.e., flexor, adductor, and internal and external rotators), especially of the shoulder flexors found to be the greatest contributor, and of elbow muscles (i.e., flexor and extensor), to generate the propulsive force. The solicitation of these muscle groups increases when accelerating the wheelchair from a complete resting position (i.e., start-up) [12] or increasing speed [13, 14]. Also of interest, few studies are pointing out the importance of voluntary trunk control in the context of manual wheelchair propulsion [9, 10, 15–17]. For large shoulder muscles (e.g., pectoralis major and latissimus dorsi) that originate from the trunk to maximally contribute to manual wheelchair propulsion, trunk strength (i.e., core stability) appears essential since no more force can be exerted on a distal segment (i.e., U/E) larger than the amount that can be counteracted proximally (i.e., trunk) to ensure seated postural stability [16]. Multidirectional seated postural stability, particularly in the sagittal plane (i.e., anteroposterior stability), is also challenged during manual wheelchair propulsion given the cyclical acceleration and deceleration to which the head, neck, and trunk segments are exposed (i.e., inertial forces) [16]. Yet, the association between U/E and trunk strength as well as seated postural stability with wheelchair propulsion performance has not been evaluated (i.e., predictive validity). Moreover, manual wheelchair propulsion performance is rarely assessed during inpatient rehabilitation, even though simple and inexpensive performance-based manual wheelchair propulsion tests (MWPTs) are available [18–23].

Upper extremity and trunk strength as well as seated postural stability, both modifiable personal characteristics through targeted rehabilitation interventions, are commonly trained during inpatient rehabilitation. Increasing strength and postural stability is expected to optimize manual wheelchair propulsion and other wheelchair-related functional abilities (i.e., concept of absolute muscular or mechanical demand). Moreover, it is also expected to minimize peripheral muscular fatigue and the risk of secondary U/E musculoskeletal impairments during manual wheelchair propulsion (i.e., concept of relative muscular or mechanical demand) [1]. Although U/E and trunk strength and seated postural stability capabilities are important contributors, only limited scientific evidence is available on specific muscle groups or trunk control inclination/perturbation directions that most closely relate to wheelchair propulsion performance. Gaining additional knowledge regarding these contributors may provide guidance to rehabilitation professionals, particularly to physical and occupational therapists, for selecting and prioritizing wheelchair selection and configurations as well as therapeutic interventions aiming to improve manual wheelchair performance, aside from those focusing on developing optimal propulsion techniques [24, 25]. Likewise, monitoring manual wheelchair propulsion performance may inform on U/E and trunk strength and seated postural stability capabilities. Moreover, manual wheelchair propulsion performance (i.e., speed) may also be a strong predictor of wheelchair skills [26, 27].

This exploratory study aimed to quantify the association between performance-based MWPTs (i.e., 20 m propulsion test, slalom test, and 6 min propulsion test), trunk and U/E strength, and seated reaching capability in individuals with a spinal cord injury (SCI) and to establish the best predictors of performance during MWPTs completed at discharge from inpatient rehabilitation. Shoulder flexor strength (i.e., greatest contributor to moment generation during wheelchair propulsion) along with forward reaching distance (i.e., best predictor of multidirectional seated postural stability) [28] were expected to be strongly associated with the performance-based MWPTs and to be among the best predictors of performance.

## 2. Methods

### 2.1. Participants

Fifteen individuals with a traumatic complete motor SCI were recruited upon discharge from a publicly-funded inpatient SCI rehabilitation program in Canada (Table 1). Individuals with a complete motor SCI were eligible to participate if they used a manually propelled wheelchair as their primary source of mobility and if their treating physical or occupational therapist confirmed that they had the ability to maintain an unsupported sitting position for at least 30 seconds and had an activity tolerance of at least 45 minutes when multiple rest periods were possible. Potential participants were excluded if they presented clinical evidence of debilitating pain or secondary musculoskeletal impairments involving their trunk or U/E, were wearing postoperative trunk orthosis or cervical brace, or had any other conditions limiting their ability to perform the MWPTs. Ethical approval was obtained from the Research Ethics Committee of the Centre for Interdisciplinary Research in Rehabilitation of Greater Montreal. Participants reviewed and signed an informed consent form before entering the study.

### 2.2. Intervention

During their publicly-funded inpatient rehabilitation stay, participants engaged in approximately one hour of physical therapy and one hour of occupational therapy for direct treatment time per day during the weekdays. During this time, participants received comparable conventional therapeutic interventions (e.g., trunk and U/E stretching and strengthening exercises, quasi-static and dynamic sitting balance exercises) articulated around a personalized treatment plan. Participants were also provided a short-term loan manual wheelchair with nonpneumatic solid rubber tires which was optimally adjusted according to specific wheelchair and seating recommendations made by the multidisciplinary rehabilitation team. Their short-term loan manual wheelchair had similar features to the ones that will be provided for long-term use upon discharge from inpatient rehabilitation. Only basic manual wheelchair skill training and propulsion technique recommendations were taught by and reviewed with the occupational therapists, respectively. All tests and measures described were completed and recorded, respectively, within 72 hours prior to discharge by a clinical research physical therapist. In parallel, demographic (i.e., age, sex, weight, height, and body mass index), clinical (i.e., American Spinal Injury Association (ASIA) impairment scale), and administrative outcome measures (i.e., time-to-admission and rehabilitation length of stay) were documented by the medical and rehabilitation professionals to characterize participants at discharge from inpatient rehabilitation.

### 2.3. Performance-Based Manual Wheelchair Propulsion Tests

After a brief familiarization period, participants completed three different tests in a random order along an unobstructed indoor smooth and levelled tiled corridor. A five-minute rest period was offered between the three tests whereas a two-minute rest period was offered between trials of each test, except for the test performed at self-selected natural velocity (i.e., 30-second rest period between trials).

#### 2.3.1. 20-Meter Propulsion Test (Figure 1(a))

Participants were instructed to propel their wheelchair at self-selected natural (20 m MWP_NAT_) and maximal velocities (20 m MWP_MAX_) from a start line until they crossed a finish line set 20 meters away. The averaged times required to complete the two 20 m MWP_NAT_ trials and the two 20 m MWP_MAX_ trials, expressed in seconds, were the main outcome measures. The 20 m MWP_NAT_ and the 20 m MWP_MAX_ have been found to be reliable (reliability indices ≥ 0.981) and precise (relative minimal detectable change = 8.5%) [29].

#### 2.3.2. Slalom Test (Figure 1(b))

Participants were instructed to propel their wheelchair at a self-selected maximum velocity along a slalom trajectory defined with 7 cones aligned in a straight line and set 3 m, 2 m, and 1 m apart from one another. The averaged time required to complete the two MWPT_SLALOM_, expressed in seconds, was the main outcome measure. The MWPT_SLALOM_ has been found to be reliable (reliability indices ≥ 0.978) and precise (relative minimal detectable change = 8.9%) [22].

#### 2.3.3. Six-Minute Propulsion Test (Figure 1(c))

Participants were instructed to propel their wheelchair along a figure 8 trajectory. While doing so, they had to propel themselves toward a cone, turn around it, and come back to the centre of the trajectory where they had to rapidly stop before repeating this sequence in the other direction. This sequence was repeated as often as possible at a self-selected maximum velocity during the six-minute propulsion period. Participants were informed of the remaining time at 2, 4, and 6 minutes. The total distance traveled, expressed in meters, recorded to the nearest meter, was the main outcome measure. The MWPT_6 min_ has been found to be highly reliable (reliability indices = 0.98) and precise (relative minimal detectable change = 7.5%) [21].

### 2.4. Upper Extremity and Trunk Impairment Measures

#### 2.4.1. Upper Extremity Strength

Maximal bilateral static strength of key U/E muscle groups (i.e., shoulder flexors, extensors, abductors, adductors, and internal and external rotators; elbow flexors and extensors; wrist flexors and extensors), expressed in Nm, was assessed in a supine position in a gravity-free plane by a single clinical research physical therapist for all participants with a microFET2 digital hand-held dynamometer (Hoggan Scientific LLC, Salt Lake City, UT) according to a standardised protocol (e.g., testing position, external stabilization, dynamometer position, and lever arm measurements) [30]. For the shoulder strength measures, the shoulder was flexed to 90° with the elbow fully extended when testing the flexors and extensors, the shoulder was in neutral position with the U/E alongside the trunk and the elbow was fully extended when testing the abductors, the shoulder was abducted to 90° with the elbow fully extended when testing the adductors, and the shoulder was in neutral position with the U/E alongside the trunk with the elbow flexed to 90° when testing the internal and external rotators. This last position was also used to measure the elbow flexor and extensor strength. Maximal bilateral handgrip strength, expressed in Kg, was assessed with a JAMAR electronic hand dynamometer (Paterson Medical, Warrenville, IL) in a sitting position with the shoulder adducted and neutrally rotated, elbow flexed at 90°, and forearm and wrist in neutral position. For U/E and handgrip strength assessments, participants had a 5-second period to progressively reach their maximal contraction and performed two trials, unless the difference between the two trials exceeded 10%, in which case a third trial was performed. Only the strongest U/E and grip strength values for each muscle group were used for all analyses. The use of quantitative measures (i.e., hand-held dynamometry) represents a better option to classify a group of individuals (i.e., discriminant construct validity) based on their U/E strength in comparison to categorical measures obtained via manual muscle testing (MMT) or the use of aggregate categorical measures (e.g., U/E score obtained with the American Spinal Injury Association (ASIA)). The ASIA U/E motor score also did not quantify handgrip strength. Hence, a change in quantitative strength measures (i.e., hand-held dynamometer), particularly for muscle groups having the ability to move their distal segments against gravity (i.e., MMT score ≥ 3/5), may not necessarily translate into a change in categorical strength (i.e., MMT) or aggregate categorical measures. Inversely, a change in categorical strength measures (i.e., MMT) or aggregate categorical measures (e.g., ASIA U/E motor score) confirms a change in quantitative strength measures (i.e., hand-held dynamometer) and related measures. Hence, the use of hand-held dynamometer is strongly encouraged in clinical practice and research protocols to measure muscle strength.

#### 2.4.2. Trunk Strength

**Figure 2:**
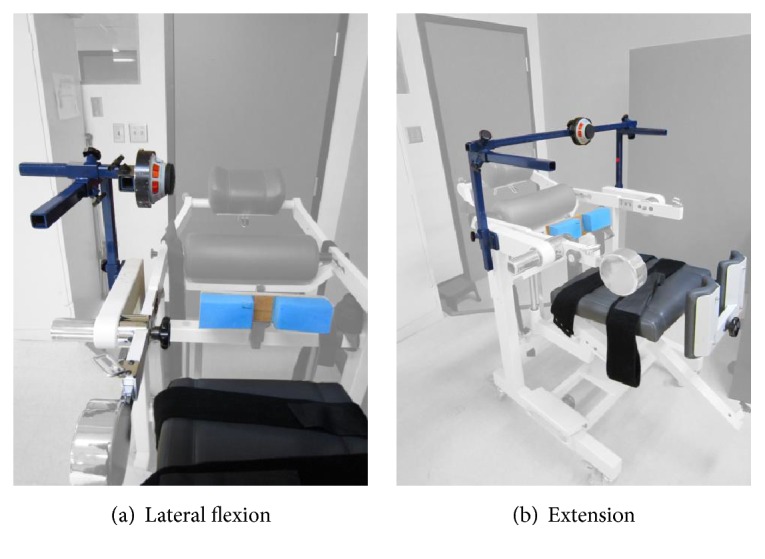
Schematic representation of the structure used to fix and stabilize the hand-held dynamometer when testing lateral flexion (a) and extension (b) of the trunk.

Maximal static trunk strength, expressed in Nm, was tested in multiple movement directions (i.e., forward flexion, lateral flexion, and extension) while sitting by a single clinical research physical therapist with a hand-held dynamometer according to a standardised protocol (e.g., testing position, external stabilization, dynamometer position, and lever arm measurement) [31]. The hand-held dynamometer was mounted on a custom-made rigid structure and positioned just below the acromion to test lateral flexion and at the 3rd thoracic vertebra to test lateral flexion and extension (Figure 2). To test forward flexion, the hand-held dynamometer was positioned over the upper part of the sternum and held by the clinical research physical therapist. Participants performed gradual maximal voluntary muscle contractions during a 5-second period (i.e., make test) and performed two trials, unless the difference between the two trials exceeded 10%, in which case a third trial was performed. The strongest strength values (i.e., moment) of each muscle group reached with each movement direction were used for all analyses. The use of a hand-held dynamometer is essential to obtain quantitative strength measures, especially since the ASIA motor score currently does not take into account the trunk strength.

#### 2.4.3. Seated Reaching Capability

Maximal seated reaching distances in five directions (i.e., forward, right and left lateral, and right and left anterolateral) were measured by a single clinical research physical therapist using a telemetric laser distance meter [31]. Maximal seated reaching distances were used as surrogate measures for seated dynamic postural stability. Participants sat over the edge of a plinth with their feet resting on the floor with about 90° knee flexion. While sitting, participants reached with their preferred hand as far as possible toward a target set at shoulder height in each tested direction, while resting the other hand on their thigh. Subjects were instructed to reach as far as possible toward the target at a self-selected velocity without losing their balance and not to stabilize their trunk with the hand resting on their thigh before returning to their initial position. The difference between the initial and furthest positions reached by a passive marker placed over the 1st thoracic vertebrae while reaching in each direction represented the maximal reaching distance. Two trials were performed in each direction and the furthest distance reached reflected the maximal seated reaching capability.

### 2.5. Statistics

Descriptive statistics (i.e., continuous data = mean ± 1 standard deviation (SD), minimum and maximum values; categorical data = proportion) were calculated for the demographic and clinical characteristics of the participants. Pearson's product-moment correlation coefficients measured the strength and direction of the proportional relationship between each MWPT separately (i.e., 20 m propulsion test, slalom test, and 6 min propulsion test) and selected clinically relevant modifiable determinants of physical impairments in the context of the present study (i.e., trunk and U/E strength as well as seated reaching capability). After inspecting each bivariate scatterplot diagram generated for potential outliers, the absolute correlation coefficient values (*r*) were interpreted according to the guidelines proposed by Altman [32]: poor (*r* < 0.20), fair (0.21–0.40), moderate (0.41–0.60), good (0.61–0.80), or very good relationship (0.81–1.00). Thereafter, separately for each MWPT, the eligible modifiable determinants (i.e., variables that correlated to *r* > 0.6 or *r* < −0.6 with a significance level of *p* ≤ 0.05) were entered into a separate stepwise linear multiple regression analysis, which combines the forward and backward selection techniques, performed for each MWPT to develop the best possible prediction equation. This analysis was selected to maximize prediction accuracy with the smallest number of predictors. A separate adjusted *R* ^2^ value was reported for each MWPT as a conservative estimate of the strength of the regression given the number of determinants considered by each model. Whenever two or more predictors were found, the degree of collinearity across predictors was assessed using the variance inflation factor (VIF) with values greater than 2.5 diagnosing a collinearity problem as it corresponds to *R* ^2^ greater than 0.60 with the other variable(s). All statistical analyses were computed with SPSS version 21.0 for Windows (SPSS, Inc., Chicago, IL).

## 3. Results

The mean (SD) of the static trunk strength, U/E strength, and seated reaching distance outcome measures as well as the MWPT outcome measures are summarized in Table 2. The correlation coefficients between the static trunk strength, U/E strength, and seated reaching test outcome measures and the performance-based timed MWPTs are summarized in Table 3. In terms of the strength-generating capability of the individual trunk muscle groups, the anterior and left lateral flexors presented a good association with the 20 m MWPT_MAX_, whereas only the left lateral flexors presented a good association with the MWPT_SLALOM_. As for the contribution of the strength-generating capability of the individual U/E muscle groups assessed on both the strongest and the weakest sides, five, six, and seven muscles groups on the strongest side and six, eight, and six muscles groups on the weakest side were found to be very good or good predictors of the 20 m MWPT_MAX_, MWPT_SLALOM_, and the MWPT_6 min_, respectively. However, only poor, fair, or moderate associations were revealed between the various U/E muscle groups assessed and the 20 m MWPT_NAT_. As for the seated reaching tests, only the forward reaching test showed a very good association with the 20 m MWPT_MAX_, MWPT_SLALOM_, and MWPT_6 min_. The best predictors selected by the regression model for each MWPT are summarized in Table 4. A total of 14, 16, and 14 determinants (i.e., possible predictor variables) were entered into the predictive modelling process for the 20 m MWPT_MAX_, MWPT_SLALOM_, and the MWPT_6 min_, respectively. The strength-generating capability of the shoulder adductors on the weakest side was the main predictor of the 20 m MWPT_MAX_, whereas the strength-generating capability of the shoulder adductors on the strongest side as well as the forward seated reaching test were the main predictors of the MWPT_SLALOM_. Although these two predictors are not completely independent, no severe collinearity problem was revealed (VIF = 1.9). As for the MWPT_6 min_, the best predictor was handgrip strength on the strongest side.

## 4. Discussion

This original exploratory study examines the association between the strength-generating capabilities of nine bilateral U/E muscle groups bilaterally, four trunk muscle groups, and five seated reaching direction capacities with the performance of MWPTs upon discharge from inpatient rehabilitation among individuals with a SCI while using a testing paradigm where there was the best potential to detect change during inpatient rehabilitation. The strength-generating capability of the shoulder adductors and handgrip muscle group as well as forward seated reaching capacity was found to best explain MWPT performance at self-selected maximum and safe velocity (i.e., 20 m MWPT_MAX_, MWPT_SLALOM_, and MWPT_6 min_). MWPT_SLALOM_ reached the highest level of the variance explained when compared to the 20 m MWPT_MAX_ and MWPT_6 min_. Specifically, the strength-generating capability of the shoulder adductors and the forward seated reaching distance were the strongest contributors and accounted for 71.3% of the variance observed during the MWPT_SLALOM_. The 20 m MWPT_NAT_ performance remains challenging to predict since the strength of the association between this test and each of the determinants studied was only low or fair and confirms that a predictive model may not allow one to pinpoint key predictors to establish priorities in clinical practice. Hence, it may be difficult to predict performance during a test completed at a self-selected natural velocity solely based on seated postural stability or trunk and U/E strength. At this velocity, it is plausible that the U/E strength and postural stability demands remain relatively low while propelling on a short distance (i.e., limited fatigue effect), which makes it difficult to pinpoint key determinants. Moreover, both manual wheelchair propulsion [33] and walking [34] at self-selected maximal velocity have been found to be significantly more responsive to change than doing so at self-selected natural velocity in individuals with SCI. Although not a focus of the present exploratory study which targets only modifiable factors during rehabilitation, the age was found to be associated to a different extent with the level of performance during the MWPT, especially during the 20 m MWPT_MAX_ and MWPT_SLALOM_. In fact, the age may have a modulating effect on seated postural stability as well as on trunk and U/E strength which, in turn, may contribute to the unexplained variance when investigating wheelchair propulsion performance.

### 4.1. Upper Extremity Strength Best Predicts the 20 m Propulsion Test

When the strength-generating capability of all muscle groups tested is examined separately, it is evident that the majority of the U/E muscle groups are associated to a different extent with the level of performance during the 20 m MWPT (especially at maximal velocity) and the two other tests performed. The shoulder flexors, adductors, internal rotators, elbow extensors, and hand/finger flexors were all found to be good or very good determinants of the tests performed at maximal velocity. This finding was expected since biomechanical studies have confirmed substantial shoulder flexion, adduction and internal rotation, along with elbow flexor and extensor contributions, during manual wheelchair propulsion [7, 11]. Among these, shoulder adductor strength on the weakest side alone was found to best predict performance during the 20 m MWPT_MAX_ and explain 53% of the observed variance. This may be explained by the fact that the key shoulder adductors (i.e., pectoralis major and latissimus dorsa muscles) originate from the trunk and attach to the humerus, allowing them to complement postural muscles and maximize trunk stability when the hands are in contact with the handrims (i.e., closed kinetic chain movements) [16]. Such synergy allows optimal forces to be applied at the handrim during propulsion since no more force can be exerted by a distal segment (i.e., U/E) than the amount that can be counteracted proximally (i.e., the trunk) to ensure stability [35, 36]. Why the shoulder adductors on the weakest side were found to be a better predictor than those on the strongest remains to be clarified in future studies. One plausible explanation for this may relate to the fact that the 20 m MWPT_MAX_ was performed along a linear trajectory on a tiled surface. In this context, the application of quasisymmetrical propulsive forces at the handrims [37] is essential to maintain the trajectory and is most likely limited by the weakest side (i.e., lowest absolute strength and highest relative demand).

### 4.2. Upper Extremity Strength and Seated Reaching Capability Best Predict the Slalom Test

Adding numerous trajectory changes occurring at maximal velocity during MWPT_SLALOM_ imposes a greater demand in terms of U/E strength and dynamic postural control in comparison to the 20 m MWPT_MAX_. In fact, the shoulder abductor strength (strong and weak sides) now becomes associated with performance, and the strength of the association is increased (+3.5–21.7%) for the U/E muscles groups that were previously associated with 20 m MWPT_MAX_. Similarly, the anterior seated reaching test is also associated to a greater extent (+13.1%) with the level of performance, in comparison, than during the 20 m MWPT_MAX_ (*r* = 0.663 versus *r* = 0.750). The anterior seated reaching and the strength of the shoulder adductor-*strongest side*, two key predictors, explain 71.3% of the observed variance. This result suggests that MWPT_SLALOM_ is most likely the most challenging test from a dynamic postural control and trunk and U/E perspectives. The trajectory changes occurring at maximal velocity generate elevated multidirectional inertial forces acting on the head-trunk-U/E segments, especially in the frontal plane (i.e., mediolateral stability), which are predominantly counteracted by two key complementary actions. First, during the anterior reaching tests, combined voluntary eccentric (forward displacement) and concentric (backward displacement) contractions of the trunk extensors (i.e., erector spinae) and of compensatory nonpostural muscles (e.g., latissimus dorsi, trapezius pars ascendens, and pectoralis major) are needed to stabilize and to position the head, trunk, and U/E segments during the tests [38, 39]. Furthermore, the fact that the anterior reaching distance, which can be administered rapidly and requires very little equipment, also provides an excellent estimation of multidirectional seated postural stability in individuals with a SCI may further explain why it was found to be a strong determinant and predictor of MWPT_SLALOM_ [28]. Second, as previously discussed, given that the shoulder adductors (i.e., pectoral major and latissimus dorsa muscles) originate from the trunk and attach to the humerus, this allows them to maximize trunk stability (i.e., closed kinetic chain movements). Theoretically, when making a rapid right turn, the inertia forces the head-trunk-U/E segments toward the left, and the right shoulder adductors counteract this effect to avoid a loss in balance. Similarly, the latissimus dorsa muscle, which also acts as a shoulder extensor, is further solicited to slow or block the right wheel to facilitate the right turn. Contrary to the 20 m MWPT_MAX_, the shoulder adductor strength on the strongest side was found to best predict performance on the MWPT_SLALOM_. This finding remains challenging to explain with a high certainty level and will deserve to be clarified in future studies. One plausible explanation of this may relate to the ipsilateral breaking force, needing to be applied rapidly and consecutively at the handrims to engage in the different turns (i.e., asymmetrical demand), that is most likely proportional to the speed at which the test is being completed.

### 4.3. Handgrip Strength Best Predicts the 6-Minute Propulsion Test

Although the U/E strength of the majority of the muscle groups assessed and the forward seated reaching distance are associated to a different extent with the MWPT_6 min_ like that previously found during the 20 m MWPT_MAX_ and MWPT_SLALOM_, the fact that the handgrip strength was selected as the best predictor (51.9% of total variance explained) is unique. This finding is plausible since handgrip strength was previously found to be a good surrogate measure to characterize overall ipsilateral U/E strength [40, 41] and a strong predictor of functional performance during activities of daily living [42] and of ambulation ability [43]. Moreover, the fact that the MWPT_6 min_ incorporates frequent stops and starts at high velocity also translates into an increased muscular demand and potential fatigability. During these tasks (i.e., stop and go), the hands need to apply substantial forces at the rims when stopping the wheelchair after each loop and starting the subsequent loop until the end of the test. These hypotheses remain to be clarified in future studies along with other key elements. Among these, the effects of handgrip and functional hand tenodesis, which are severly impaired in many individuals with complete motor high-level tetraplegia, will deserve additional attention. It is anticipated that the performance on the MWPT_6 min_ may become difficult to predict among these individuals since the propulsion technique, particularly the application of the propulsive and breaking forces to the handrims, may not require a handgrip any longer (e.g., palmar technique,and handrim modifications). Moreover, the altered autonomic responses affecting individuals with tetraplegia also need to be considered when administering the MWPT_6 min_.

### 4.4. Study Limitations

In the context of this exploratory study, the small sample size confirms the relevance of the constructs investigated (i.e., trunk control via multidirectional seated reaching tests and trunk and U/E strength) but uncertainties about the best predictor(s) continue. A large confirmatory study with a sample size of about 150 participants is needed to strengthen the current results considering, for example, the 10 : 1 sample size estimation rule of thumb (i.e., an effective sample size of 10 participants per determinant examined). Because the study only included manual wheelchair users with recent SCI undergoing an initial intensive rehabilitation phase in a publicly funded healthcare system, the generalizability of the results beyond this reference population also requires caution. Nonetheless, and in spite of the variability observed across manual wheelchair users included in the present study, the results support the relevance to provide U/E and trunk strengthening and dynamic sitting balance training in rehabilitation programs. Moreover, the results support the need to gain additional insight into the most effective rehabilitation strategies to optimize U/E and trunk strength and dynamic sitting balance recovery and their effects on performance during manual wheelchair propulsion among a large cohort of manual wheelchair users with a recent SCI within an inpatient multidisciplinary SCI rehabilitation program. Other complementary rehabilitation strategies targeting wheelchair types and configurations (e.g., horizontal and vertical rear axle positions relative to the shoulder joint position, seat tilt, type of lateral supports, and backrest) as well as propulsion techniques (e.g., movement strategies and mechanical effectiveness of handrim force application) may also deserve additional attention in future studies, especially since a substantial proportion of the variance of the MWPTs still remained unexplained (i.e., ≥28.7%). Moreover, the fact that the determinants and predictors in the present study solely focused on some potentially modifiable personal physical factors during inpatient rehabilitation (e.g., U/E strength and seated postural stability) may also need consideration since other nonphysical or nonmodifiable factors may need to be considered in the future (e.g., sex, level of injury, and time since injury). Prudence is also suggested when inferring from the present results as no valid assumptions about causative factors can be made solely on the present results. Last, in terms of a comprehensive assessment of manual wheelchair performance, combining these performance-based tests with a manual wheelchair skill assessment will also be warranted in the future.

## 5. Conclusion

The trunk and U/E strength-generating capability, especially of the shoulder adductors, and forward seated reaching capacity are key determinants of performance during MWPTs upon discharge from rehabilitation among individuals with a SCI. Rehabilitation interventions targeting these determinants should be encouraged in clinical practice to optimize performance during manual wheelchair propulsion. The use of distance- or time-based MWPTs performed at self-selected maximal velocity is advised in clinical practice or research protocols.

## Figures and Tables

**Figure 1:**
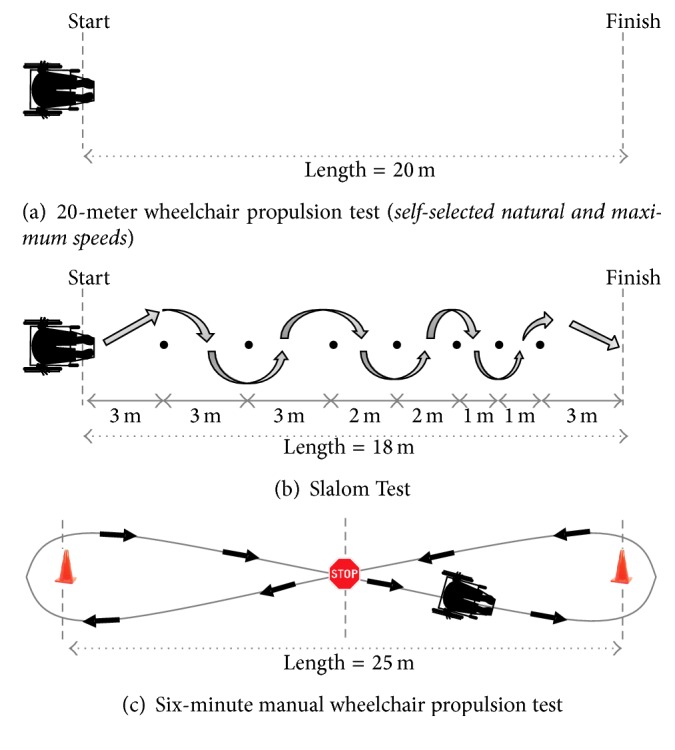
Schematic drawings of the manual wheelchair propulsion tests.

**Table 1:** Demographic, clinical, and administrative outcome measures describing participants (*N* = 15).

Outcome measure	Mean (±1SD)	[Min–max]
Age (yrs)	32.7 (9.4)	[23.2–58.5]
Sex (male/female)	14/1	
Height (m)	1.77 (0.12)	[1.42–1.91]
Weight (kg)	76.6 (13.0)	[59.1–102.7]
Body mass index (m/kg^2^)	24.6 (4.4)	[18.3–32.3]
ASIA neurological level (range)	C8-T12	
ASIA impairment score (AIS)		
*Motor-total*	48.4 (11.6)	[18–69]
*Motor-U/E*	45.9 (9.5)	[18–50]
*Motor-L/E*	2.5 (5.4)	[0–21]
*Sensory-total*	108.2 (53.5)	[33–182]
*Sensory-light touch*	55.4 (27.7)	[17–99]
*Sensory-pinprick*	52.8 (25.9)	[160–84]
Rehabilitation length of stay (days)	75.3 (22.0)	[46–127]
Time to rehabilitation admission (days)	32.7 (18.9)	[11–87]

**(a):** 

Wheelchair performance tests	Mean (SD)	[Min; max]
Timed 20 m propulsion test (s)		
*Natural velocity *	15.67 (2.47)	[10.99–20.01]
*Maximal velocity *	10.16 (2.14)	[8.43–16.36]
Timed slalom test (s)	18.55 (4.63)	[15.21; 29.42]
6 min propulsion test (m)	518.5 (121.7)	[256.5; 771.5]

Static trunk strength (Nm/kg)	Mean (SD)	[Min–max]

Anterior flexion	0.28 (0.18)	[0.03–0.58]
Extension	0.36 (0.22)	[0.10–0.85]
Right lateral flexion	0.27 (0.10)	[0.09–0.44]
Left lateral flexion	0.31 (0.11)	[0.13–0.56]

Seated reaching test (cm)	Mean (SD)	[Min–max]

Anterior	39.31 (17.69)	[3.60–66.29]
Right lateral	9.48 (4.41)	[1.90–16.55]
Left lateral	8.98 (4.14)	[3.67–17.9]
Right anterolateral	12.49 (10.19)	[1.60–37.68]
Left anterolateral	12.11 (11.04)	[3.40–51.80]

**(b):** 

Upper extremity strength (Nm/kg)	Strongest side		Weakest side	
	Mean (SD)	[Min–max]	Mean (SD)	[Min; max]
Shoulder				
Flexors	0.73 (0.14)	[0.45–1.00]	0.67 (0.15)	[0.34–0.93]
Extensors	0.96 (0.25)	[0.33–1.23]	0.87 (0.26)	[0.22–1.14]
Abductors	0.91 (0.21)	[0.57–1.43]	0.82 (0.19)	[0.52–1.31]
Adductors	0.83 (0.25)	[0.24–1.10]	0.71 (0.23)	[0.15–1.00]
External rotators	0.52 (0.14)	[0.25–0.74]	0.43 (0.09)	[0.19–0.58]
Internal rotators	0.65 (0.22)	[0.24–1.02]	0.52 (0.17)	[0.13–0.79]
Elbow				
Flexors	0.92 (0.24)	[0.53–1.31]	0.84 (0.24)	[0.48–1.19]
Extensors	0.56 (0.23)	[0.00–0.82]	0.49 (0.21)	[0.00–0.81]
Handgrip (kg)	41.0 (20.2)	[0.00–70.2]	36.8 (19.4)	[0.00–69.8]

**Table 3:** Pearson product-moment correlation coefficient (*r*) between the clinical, static trunk strength, seated reaching test, and U/E strength outcome measures versus the performance-based timed manual wheelchair tests. For each wheelchair performance test, correlation coefficients highlighted in bold represent the variable entered in its multiple regression analysis (i.e., *r* > 0.6 or *r* < −0.6). Note that the proportion of the variance (*R* ^2^) in the wheelchair performance tests that is predictable from each independent variables can be computed by squaring the *r* value (*r* ^2^) reported in the present table.

	Wheelchair performance tests			
	Timed 20 m propulsion test (s)		Timed slalom test (s)	6 min propulsion test (m)
	*Natural velocity*	*Maximal velocity*		
*Clinical variables*				
Age (yrs)	0.599^*∗*^	0.639^*∗∗*^	0.635^*∗*^	−0.504
Height (m)	0.010	0.227	0.293	−0.263
Weight (kg)	−0.371	−0.086	0.037	−0.213
Body mass index (m/kg^2^)	−0.339	−0.244	−0.189	−0.021

*Static trunk strength (Nm/kg)*				
Anterior flexion	−0.595^*∗*^	−0.625^*∗*^	−0.581^*∗*^	0.337
Extension	−0.523^*∗*^	−0.465	−0.423	0.254
Right lateral flexion	−0.287	−0.581^*∗*^	−0.559^*∗*^	0.358
Left lateral flexion	−0.419	−0.623^*∗*^	−0.631^*∗*^	0.348

*Seated reaching test (cm)*				
Forward	−0.511	−0.663^*∗∗*^	−0.750^*∗∗*^	0.622^*∗*^
Right lateral	−0.283	−0.537^*∗*^	−0.551^*∗*^	0.397
Left lateral	−0.269	−0.338	−0.371	0.413
Right anterolateral	0.054	−0.383	−0.408	0.235
Left anterolateral	−0.228	−0.094	−0.161	0.095

*Upper extremity strength (Nm/kg)*				
Shoulder				
Flexors				
Strongest side	−0.221	−0.655^*∗*^	−0.715^*∗∗*^	0.669^*∗∗*^
Weakest side	−0.266	−0.719^*∗∗*^	−0.773^*∗∗*^	0.685^*∗∗*^
Extensors				
Strongest side	−0.441	−0.681^*∗∗*^	−0.756^*∗∗*^	0.629^*∗*^
Weakest side	−0.366	−0.665^*∗∗*^	−0.722^*∗∗*^	0.618^*∗*^
Abductors				
Strongest side	−0.195	−0.512	−0.623^*∗*^	0.503
Weakest side	−0.234	−0.560^*∗*^	−0.651^*∗*^	0.469
Adductors				
Strongest side	−0.360	−0.744^*∗∗*^	−0.806^*∗∗*^	0.709^*∗∗*^
Weakest side	−0.414	−0.752^*∗∗*^	−0.804^*∗∗*^	0.612^*∗*^
External rotators				
Strongest side	−0.175	−0.466	−0.551^*∗*^	0.634^*∗*^
Weakest side	−0.349	−0.639^*∗*^	−0.662^*∗∗*^	0.626^*∗*^
Internal rotators				
Strongest side	−0.007	−0.393	−0.456	**0.600**
Weakest side	−0.161	−0.557^*∗*^	−0.548^*∗*^	0.496
Elbow				
Flexors				
Strongest side	−0.074	−0.488	−0.573^*∗*^	0.489
Weakest side	−0.092	−0.527	−0.630^*∗*^	0.585^*∗*^
Extensors				
Strongest side	−0.269	−0.654^*∗*^	−0.727^*∗∗*^	**0.649**
Weakest side	−0.409	−0.684^*∗∗*^	−0.769^*∗∗*^	0.645^*∗*^
Handgrip				
Strongest side	−0.436	−0.735^*∗∗*^	−0.794^*∗∗*^	0.749^*∗∗*^
Weakest side	−0.474	−0.674^*∗∗*^	−0.719^*∗∗*^	0.698^*∗∗*^

^*∗*^Significance set at *p* < 0.05; ^*∗∗*^significance set at *p* < 0.01.

**Table 4:** Key predictors of the performance-based timed manual wheelchair tests.

Regression equation	Adjusted *R* ^2^	Beta	*p* value
Timed 20 m propulsion test (s)			
*Natural velocity = *	No attempt to generate a predictive model		
*Maximal velocity =*	0.530		
*15.233+*			
−*7.003* ^*∗*^ * shoulder adductor-weakest side*		−0.752	0.002

^*∗*^Timed slalom test (s) =	0.713		
30.880+			
−9.046^*∗*^ shoulder adductors- strongest side+		−0.480	0.043
−11.561^*∗*^ seated reaching test-forward		−0.463	0.048

6 min propulsion test (m) =	0.519		
332.898+			
4.492 handgrip-strongest side		0.749	0.002

^*∗*^Variable inflation factor = 1.9.
